# Neural Mechanism of Facilitation System during Physical Fatigue

**DOI:** 10.1371/journal.pone.0080731

**Published:** 2013-11-20

**Authors:** Masaaki Tanaka, Akira Ishii, Yasuyoshi Watanabe

**Affiliations:** 1 Department of Physiology, Osaka City University Graduate School of Medicine, Osaka, Japan; 2 RIKEN Center for Life Science Technologies, Kobe, Japan; Inserm, France

## Abstract

An enhanced facilitation system caused by motivational input plays an important role in supporting performance during physical fatigue. We tried to clarify the neural mechanisms of the facilitation system during physical fatigue using magnetoencephalography (MEG) and a classical conditioning technique. Twelve right-handed volunteers participated in this study. Participants underwent MEG recording during the imagery of maximum grips of the right hand guided by metronome sounds for 10 min. Thereafter, fatigue-inducing maximum handgrip trials were performed for 10 min; the metronome sounds were started 5 min after the beginning of the handgrip trials. The metronome sounds were used as conditioned stimuli and maximum handgrip trials as unconditioned stimuli. The next day, they were randomly assigned to two groups in a single-blinded, two-crossover fashion to undergo two types of MEG recordings, that is, for the control and motivation sessions, during the imagery of maximum grips of the right hand guided by metronome sounds for 10 min. The alpha-band event-related desynchronizations (ERDs) of the motivation session relative to the control session within the time windows of 500 to 700 and 800 to 900 ms after the onset of handgrip cue sounds were identified in the sensorimotor areas. In addition, the alpha-band ERD within the time window of 400 to 500 ms was identified in the right dorsolateral prefrontal cortex (Brodmann's area 46). The ERD level in the right dorsolateral prefrontal cortex was positively associated with that in the sensorimotor areas within the time window of 500 to 700 ms. These results suggest that the right dorsolateral prefrontal cortex is involved in the neural substrates of the facilitation system and activates the sensorimotor areas during physical fatigue.

## Introduction

Fatigue can be defined as difficulty in initiating or sustaining voluntary activities [1]. Fatigue can be classified as physical or mental, and physical fatigue can be classified as peripheral or central. In contrast to peripheral fatigue, central fatigue is caused at sites proximal to the peripheral nerves and is defined as a progressive decline in the ability to activate muscles voluntarily [2], [3].

Recently, we performed a neuroimaging study of classical conditioning of physical fatigue [4]. Participants underwent magnetoencephalography (MEG) measurements during the imagery of maximum handgrips guided by metronome sounds for 10 min. Thereafter, fatigue-inducing physical task trials were performed for 10 min; metronome sounds were started 5 min after the beginning of the task trials. The metronome sounds were used as conditioned stimuli and the physical task trials were used as unconditioned stimuli to cause central fatigue. The next day, neural activities during the imagery of maximum handgrips guided by metronome sounds for 10 min were measured. The level of fatigue sensation caused by listening to the metronome sounds on the second day was increased relative to the first day and the equivalent current dipoles (ECDs) in the insular cortex and posterior cingulate cortex were observed only after the conditioning session. These MEG results showed that classical conditioning of physical fatigue took place, and that these brain regions were involved in the neural substrates of central fatigue, which limits the motor output from the primary motor cortex (M1) and physical performance under the condition of physical fatigue.

A motivational input activates a facilitation system to increase the motor output from M1 to overcome central fatigue [5]. The frontal area, in particular the dorsolateral prefrontal area, seems to play a pivotal role in increasing motor output, that is, enhancing the facilitation system, against the effects of central fatigue. After a fatigue-inducing physical task, an increased movement-evoked MEG response to the imagery of maximum handgrips in the dorsolateral prefrontal area was shown and the activation level in this brain area was positively associated with those in the bilateral sensorimotor areas [6]. Because the endurance duration for a physical task was shown to be related to activation in the prefrontal area [7], the prefrontal area seems to contribute to activation in the bilateral sensorimotor areas to compensate for central fatigue. We therefore hypothesized that the dorsolateral prefrontal cortex is involved in the neural substrates of the facilitation system during physical fatigue. However, to confirm that the dorsolateral prefrontal cortex is related to the facilitation system against central fatigue, it is essential to investigate the neural substrates under conditions without physical fatigue while participants feel central inhibition [8], that is, fatigue sensation, such as a conditioned physical fatigue. This is because physical fatigue is a complex phenomenon or state, and confounding factors other than central inhibition and the facilitation system may be involved [5], [9].

The aim of the present study was to identify the neural mechanisms of the facilitation system during physical fatigue. We compared neural activities between the motivation and unmotivated control conditions under the condition of classical conditioning of physical fatigue. In addition to having a high temporal resolution, MEG has an advantage of measuring brain activity by using time-frequency analyses [10]. Oscillatory brain rhythms are considered to originate from synchronous synaptic activities of a large number of neurons [11]. Synchronization of neural networks may reflect integration of information processing, and such synchronization processes can be evaluated using MEG time-frequency analyses. Multiple, broadly distributed, and continuously interacting dynamic neural networks are achievable through the synchronization of oscillations at particular time-frequency bands [12]. In particular, event-related desynchronization (ERD) of alpha (8–13 Hz) and beta (13–25 Hz) frequency bands were reported to be associated with fatigue in the central nervous system [13]–[15]. Alterations of the decreased MEG alpha and beta power densities in some brain regions induced by enhanced motivation during the imagery of maximum handgrips may provide valuable clues to identifying neural mechanisms of the facilitation system under the condition of physical fatigue. In addition, the correlation analyses among the MEG variables may provide important clues regarding the roles of MEG variables in physical fatigue. Therefore, correlation analyses were conducted to evaluate the relationships among the MEG responses.

## Methods

### Participants

Twelve healthy male volunteers (age, 24.8±7.2 years [mean ± SD]) were enrolled. According to the Edinburgh handedness inventory [16], all participants were right-handed. Current smokers, participants with a history of mental or brain disorders, and those taking chronic medications that affect the central nervous system were excluded. All the participants provided written informed consent before participation. This study was approved by the Ethics Committee of Osaka City University and was conducted in accordance with the principles of the Declaration of Helsinki. Minors or children are not involved in the participants of our study.

### Experimental design

**Figure 1:**
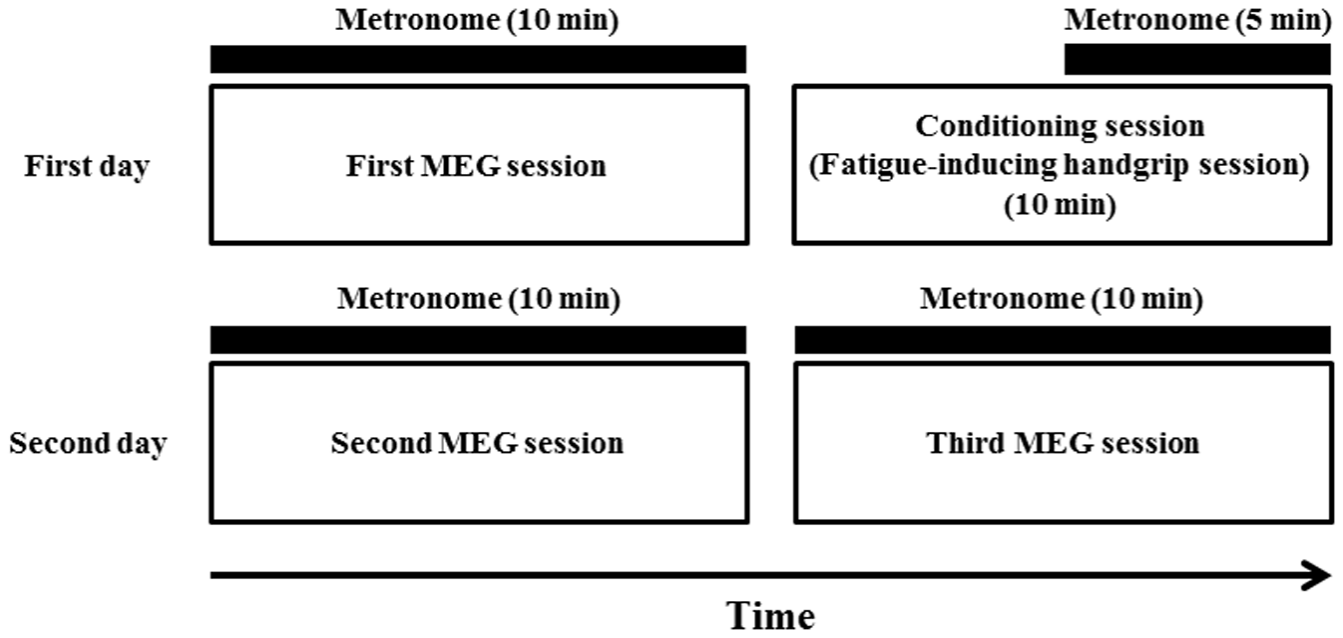
Experimental design. Participants were randomly assigned to two groups in a single-blinded, two-crossover fashion to perform two types of magnetoencephalography (MEG) sessions (the same participants performed control and motivation sessions and the order of the sessions was counterbalanced, that is, six participants began with the control session and the others began with the motivation session). On the first day, neural activities during the imagery of handgrips guided by the handgrip cues of metronome sounds were measured using MEG for 10 min (first MEG session). Thereafter, 10-min fatigue-inducing maximum handgrip trials (conditioning session) were performed, in which metronome sounds were started 5 min after the beginning of the handgrip trials. The metronome sounds were used as conditioned stimuli and maximum handgrip trials as unconditioned stimuli. On the next day, two 10-min MEG recordings during the imagery of handgrips guided by the handgrip cues of metronome sounds were performed (second and third MEG sessions, that is, the control and motivation sessions).

The experiment consisted of three MEG sessions and a single conditioning session (Fig. 1). After enrollment, participants were randomly assigned to two groups in a single-blind, two-crossover fashion to perform two types of MEG sessions: a control session and a motivation session. The same participants performed the control and motivation sessions and the order of the sessions was counterbalanced, that is, six participants began with the control session and the others began with the motivation session. On the first day, MEG recordings during the imagery of maximum grips of the right hand, guided by metronome sounds, was performed for 10 min (first MEG session). Thereafter, 10-min fatigue-inducing maximum handgrip trials using a device (HAND GRIPS 30 kg; IGNIO, Nagoya, Japan) were performed (conditioning session), in which the metronome sounds (same as the MEG session) were started 5 min after the beginning of the handgrip trials and the sounds were continued until the end of the handgrip trials. The metronome sounds were used as the conditioned stimuli and maximum handgrip trials as the unconditioned stimuli to cause fatigue sensation [4]. Participants were not informed about the metronome sounds before the task trials. On the next day, two 10-min MEG recordings during the imagery of maximum grips of the right hand guided by metronome sounds were performed (second and third MEG sessions). In the motivation session, participants were instructed that when they performed the imagery of maximum grips of the right hand with the maximum level of motivation, they could earn up to 7000 yen (approximately $70). In the control session, they could not obtain any monetary rewards even when they performed the imagery with the maximum level of motivation. Participants were told that electrocardiography was being performed and recorded during the MEG sessions to assess motivation levels.

Each MEG session consisted of 150 blocks, and each block consisted of three pacing cues followed by one handgrip cue. During the MEG session, participants heard the sound cues every 1 s with their eyes closed, and every 4 s during the handgrip cue period, they were requested to imagine that they were gripping a soft ball with their right hand at a maximal voluntary contraction level for 1 s. The pacing cue consisted of white noise that lasted 33 ms; the handgrip cue consisted of a 1000 Hz tone that lasted 1 s. All the cue sounds were produced by Windows Media Player (Microsoft Corporation, Redmond, WA) and were converted to electric signals by a sound card (Creative X-Fi Audio Processor [WDM]; Creative Technology, Singapore, Singapore) installed in a personal computer (DELL Precision 390; Dell, Round Rock, TX). The sound signal was amplified by an audio amplifier (MA-500U; Onkyo Corporation, Tokyo, Japan) outside of the magnetically shielded room.

During the conditioning session, participants watched a fixed mark (+; black mark on a white background) on a screen placed in front of their eyes using a video projector (PG-B10S; SHARP, Osaka, Japan). When a handgrip cue mark (×; black mark on white background) was presented instead of the fixation mark every 4 s, they were requested to perform a handgrip with their right hand at a maximal voluntary contraction level for 1 s by gripping the device. The timing of the visual handgrip cues was the same as that of the metronome handgrip cue sounds that started 5 min after the beginning of the handgrip trials.

Just before and after the conditioning session, participants were asked to subjectively rate the fatigue level of the right and left hands using a visual analogue scale (VAS) ranging from 0 (minimum) to 100 (maximum) [17]. In addition, just after the control and motivation sessions, VAS scores for motivation were measured.

This study was conducted in a quiet, temperature-, and humidity-controlled, magnetically shielded room. During the experiment, participants lay on a bed in the supine position. For 1 day before each visit, participants refrained from intense physical and mental activities and caffeinated beverages, consumed a normal diet, and maintained normal sleeping hours.

### MEG recordings

MEG recordings were performed using a 160-channel whole-head type MEG system (MEG vision; Yokogawa Electric Corporation, Tokyo, Japan) with a magnetic field resolution of 4 fT/Hz^1/2^ in the white-noise region. The sensor and reference coils were gradiometers 15.5 mm in diameter and 50 mm at baseline, and each pair of sensor coils was separated at a distance of 23 mm. The sampling rate was 1000 Hz with a 200 Hz hard low-pass filter and a 0.3 Hz hard high-pass filter.

### MEG data analyses

MEG signal data were analyzed offline after analogue-to-digital conversion. Magnetic noise originating from outside the shield room was eliminated by subtracting the data obtained from reference coils using a software program (MEG 160; Yokogawa Electric Corporation) followed by artifact rejection by careful visual inspection. The MEG data were split into segments of 1000 ms length (from 0 to 1000 ms after the onset of each handgrip cue sound), and the segments were averaged. After averaging, data were band-pass filtered by a fast Fourier transform using Frequency Trend (Yokogawa Electric Corporation) to obtain time-frequency band signals using a software Brain Rhythmic Analysis for MEG (BRAM; Yokogawa Electric Corporation) [18].

Localization and intensity of the time-frequency power of cortical activities were estimated using BRAM software, which used narrow-band adaptive spatial filtering methods as an algorithm [18]. These data were then analyzed using statistical parametric mapping (SPM8, Wellcome Department of Cognitive Neurology, London, UK), implemented in Matlab (Mathworks, Sherbon, MA). The MEG anatomical/spatial parameters used to warp the volumetric data were transformed into the Montreal Neurological Institute (MNI) template of T1-weighed images [19]) and applied to the MEG data. The anatomically normalized MEG data were filtered with a Gaussian kernel of 20 mm (full-width at half-maximum) in the x, y, and z axes (voxel dimension was 5.0×5.0×5.0 mm). The decreased oscillatory power, that is, ERD, for alpha (8–13 Hz) and beta (13–25 Hz) bands within the time window of 0 to 1000 ms (every 100 ms) in the motivation session relative to the control session was measured on a region-of-interest basis to obtain the neural activation pattern of the facilitation system during physical fatigue. The resulting set of voxel values for each comparison constituted a SPM of the t statistics (SPM{T}). The threshold for the SPM{T} of individual analyses was set at P<0.05 (corrected for multiple comparisons). The weighted sum of the parameters estimated in the individual analyses consisted of “contrast” images, which were used for the group analyses [20]. Individual data were summarized and incorporated into a random-effect model so that inferences could be made at a population level [20]. SPM{T} for the contrast images were created as described above. Significant signal changes for each contrast were assessed by means of t statistics on a voxel-by-voxel basis [20]. The threshold for the SPM{T} of group analyses was set at P<0.05 (corrected for multiple comparisons). Anatomical localizations of significant voxels within clusters were done using the Talairach Demon software [21].

### Magnetic resonance imaging overlay

Anatomic magnetic resonance imaging (MRI) was performed using a Philips Achieva 3.0TX (Royal Philips Electronics, Eindhoven, The Netherlands) for all participants to permit registration of magnetic source locations with their respective anatomic locations. Before MRI scanning, five adhesive markers (Medtronic Surgical Navigation Technologies Inc., Broomfield, CO) were attached to the skin of each participant's head (the first and second ones were located 10 mm anterior the left tragus and right tragus, the third at 35 mm superior the nasion, and the fourth and fifth at 40 mm right and left of the third one). MEG data were superimposed on MRI scans using information obtained from these markers and MEG localization coils.

### Statistical analyses

Values are presented as mean ± SD, unless otherwise stated. Two-way analyses of variance (ANOVA) for repeated measures were performed to assess the effects of the hand (right or left) and time course within the conditioning session on the subjective level of fatigue. The paired t-test was used to evaluate significant differences between two conditions. Pearson's correlation analyses were conducted to evaluate the relationships between two MEG responses. In the analyses, the number of cases varied due to incidental missing values. All P values were two-tailed, and values less than 0.05 were considered statistically significant. Statistical analyses were performed using IBM SPSS 20.0 (IBM, Armonk, NY).

## Results

**Figure 2:**
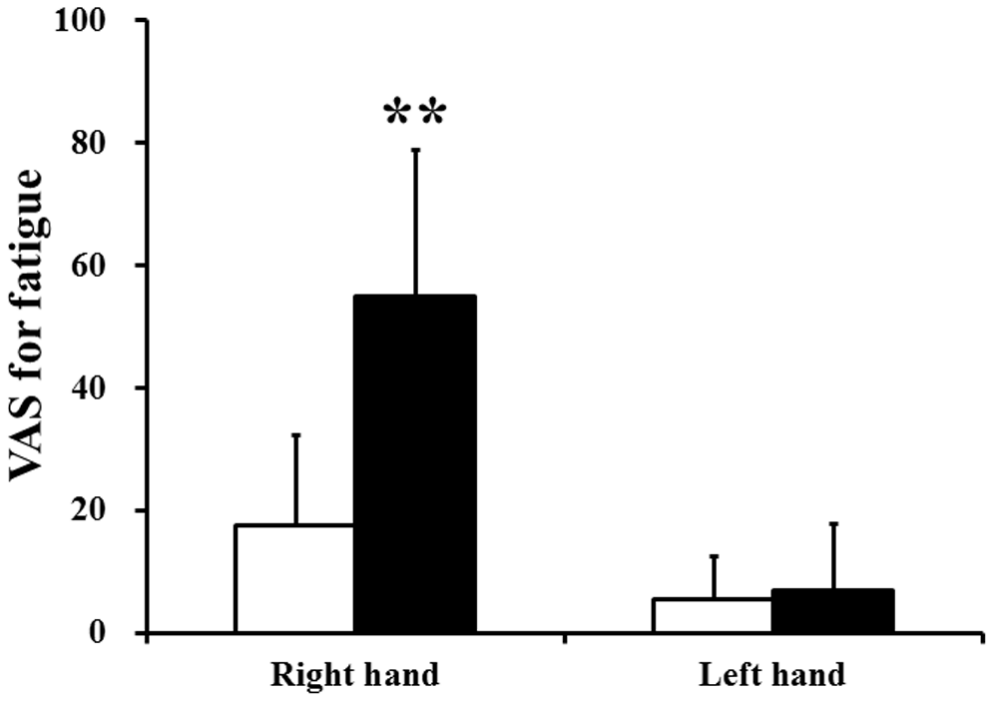
Visual analogue scale (VAS) values of right and left hands for fatigue immediately before (open columns) and after (closed columns) the 10-min fatigue-inducing handgrip trials. Data are mean and SD. **P<0.01, significantly different from the corresponding values before the fatigue-inducing trials (paired t-test).

**Figure 3:**
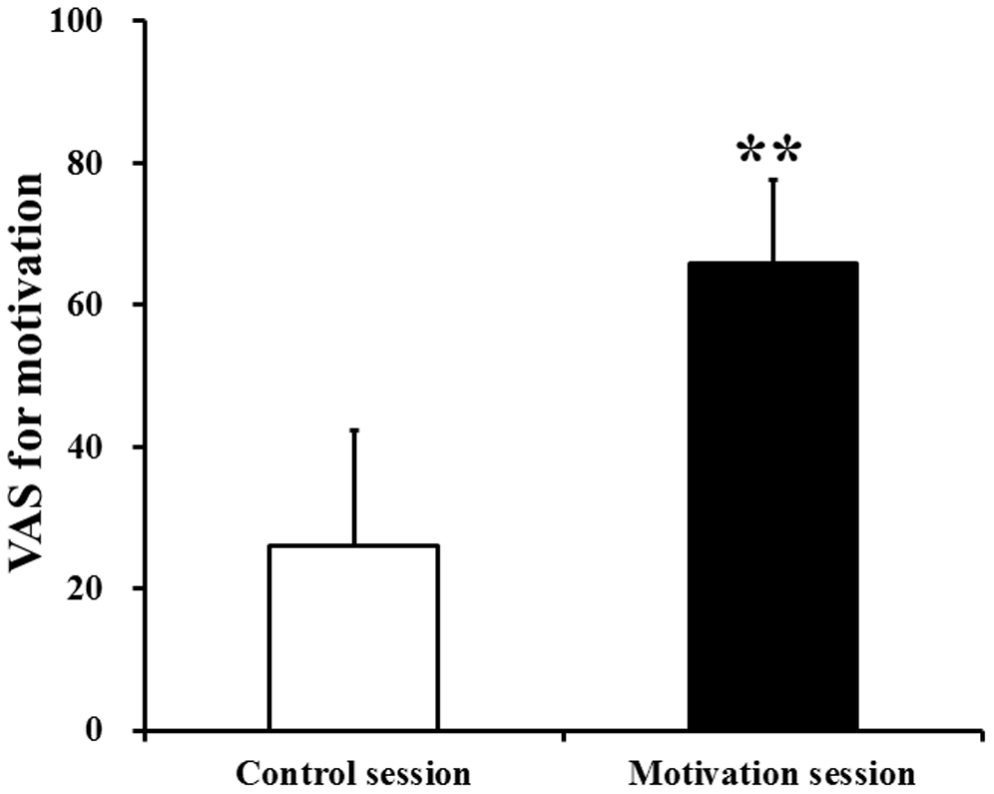
Visual analogue scale (VAS) values for motivation during the control session (open column) and the motivation session (closed column). Data are mean and SD. **P<0.01, significantly different from the corresponding values of the control session (paired t-test).

To assess the changes in the subjective level of fatigue after the 10-min maximum handgrip trials, two-way ANOVAs for repeated measures were performed. Significant main effects of hand [F(1,11) = 64.49, P<0.001] and time course [F(1,11) = 25.24, P<0.001] and a hand × time course interaction effect [F(1,11) = 22.97, P = 0.001] on VAS scores were shown. The level of subjective fatigue of the right hand after the handgrip trials was significantly higher than that before the handgrip trials (Fig. 2). However, the level of subjective fatigue of the left hand was not altered after the handgrip trials (Fig. 2). We also assessed the difference in the subjective level of motivation between the control and motivation sessions. The subjective level of motivation during the motivation session was significantly higher than during the control session (Fig. 3).

**Figure 4:**
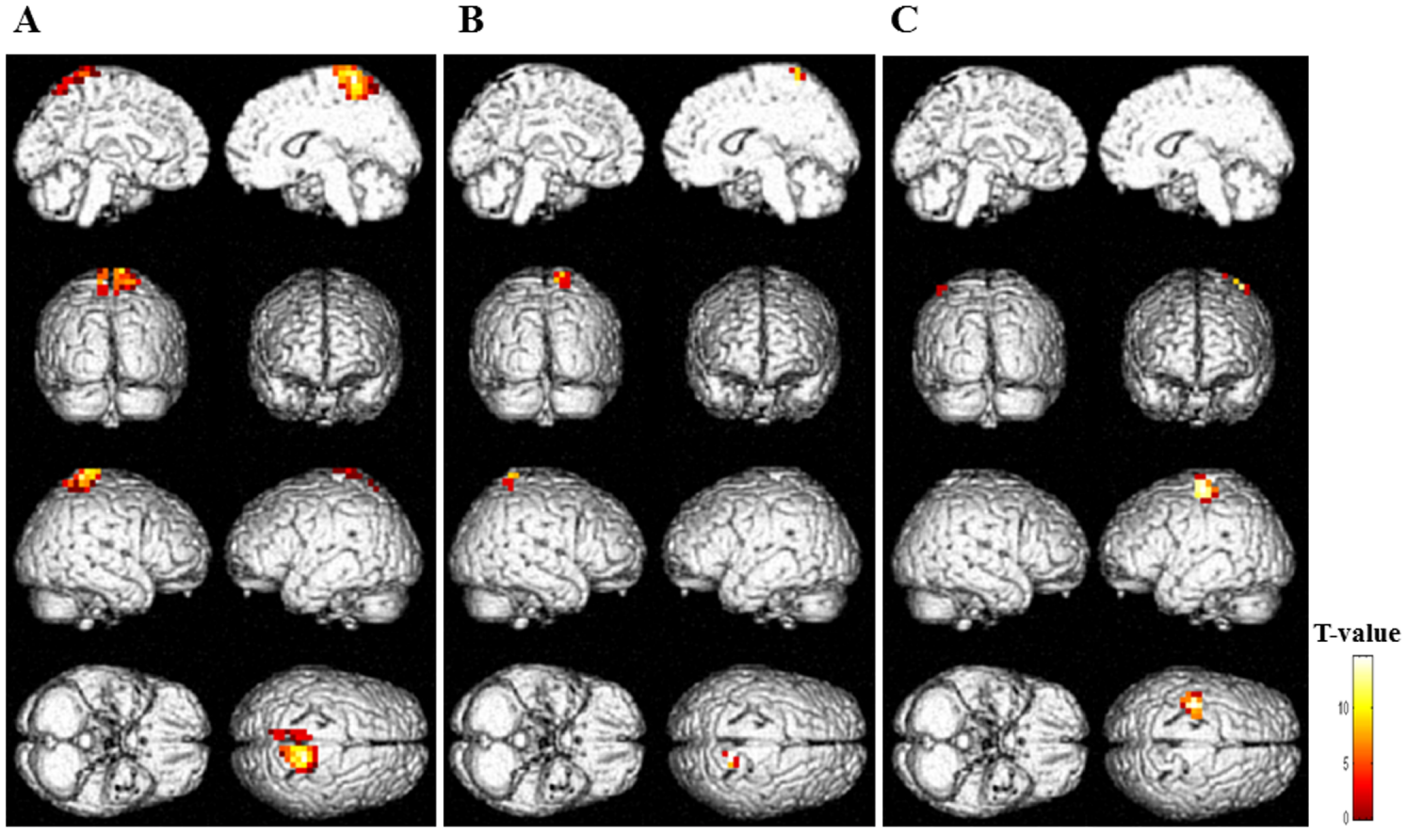
Statistical parametric maps of event-related desynchronization (the motivation session relative to the control session; random-effect analyses of 12 participants, P<0.05, corrected for multiple comparisons at the voxel level) of alpha frequency band in the right postcentral gyrus (time window of 500–600 ms after the onset of handgrip cue sounds) (A), left postcentral gyrus (500–600 ms) (B), right postcentral gyrus (600–700 ms) (C), and left precentral gyrus (800–900 ms) (D). Statistical parametric maps are superimposed on surface-rendered high-resolution MRIs. The color bar indicates T-values.

To identify the brain regions associated with the facilitation system during physical fatigue, the decreased oscillatory power, that is, ERD, for alpha and beta frequency bands in the motivation session relative to the control session was assessed. These results are shown in Table 1 and Figure 4. As for the alpha frequency band, in the time windows of 500 to 600 ms (Figure 4A) and 600 to 700 ms (Figure 4B) after the onset of handgrip cue sounds in the right sensorimotor area and time windows of 500 to 600 (Figure 4A) and 800 to 900 ms (Figure 4C) in the left sensorimotor areas, the ERDs were identified (P<0.05, corrected for multiple comparisons at the voxel level), in the right sensorimotor area and left sensorimotor area, respectively. As for the beta frequency band, no significant ERDs were identified within any of the time windows.

**Table 1:** Brain regions that showed event-related desynchronization of alpha frequency band in the motivation session relative to the control session.

Location	Reaction time (ms)	Brodmann's area	Coordinate (mm)			T-value
			x	y	z	
Postcentral gyrus	500–600	3	17	−42	75	9.07
Postcentral gyrus	500–600	7	−3	−42	80	6.86
Postcentral gyrus	600–700	3	17	−47	75	6.41
Precentral gyrus	800–900	4	−33	−22	65	7.79

x, y, z: Stereotaxic coordinates of peak of activated clusters.

Random-effect analyses of 12 participants (*P*<0.05, corrected for multiple comparisons at voxel levels).

**Figure 5:**
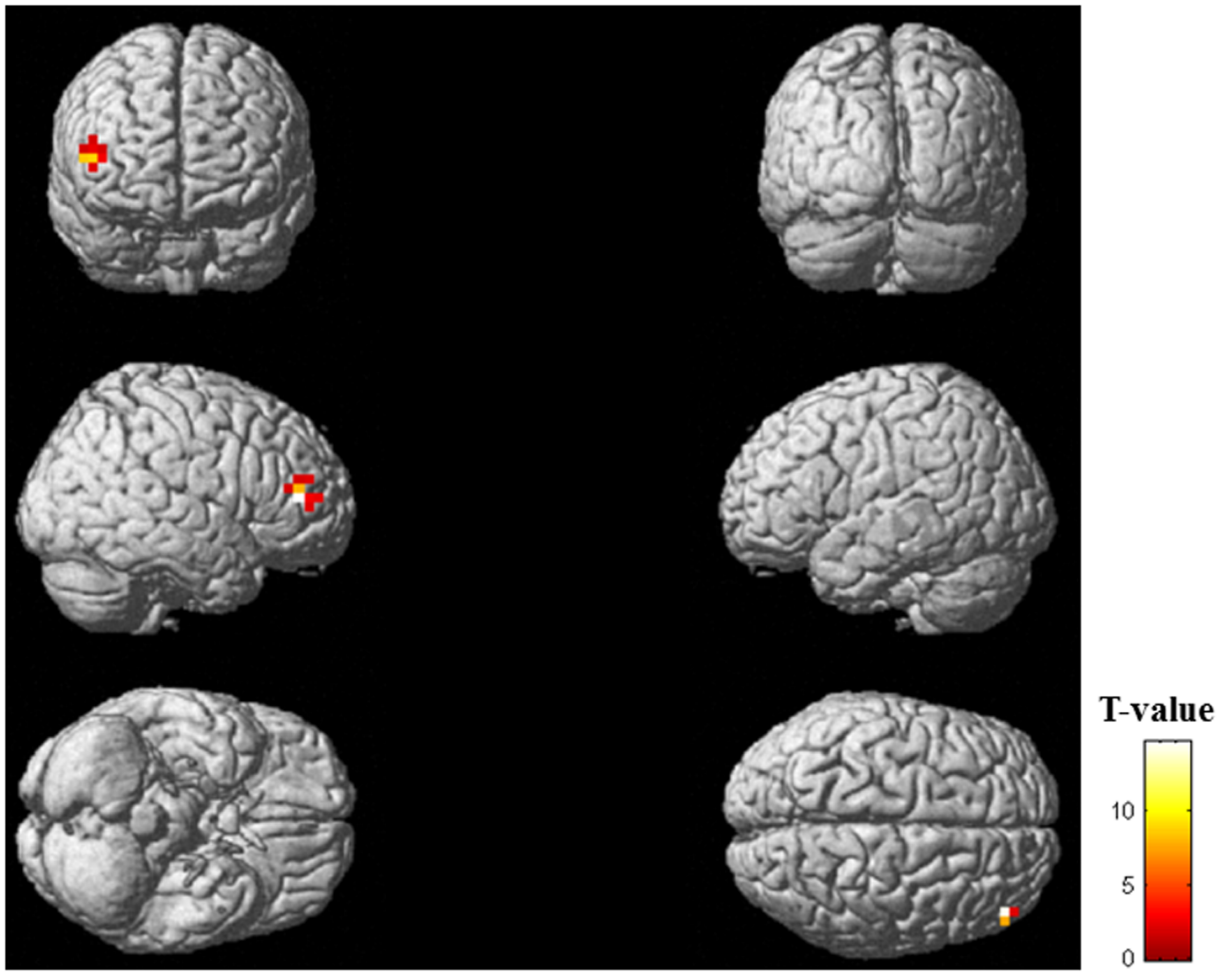
Statistical parametric maps of event-related desynchronization (the motivation session relative to the control session; random-effect analyses of 12 participants, P<0.05, corrected for multiple comparisons at the cluster level) of alpha frequency band in the inferior frontal gyrus (Brodmann's area 46) within the time window of 400 to 500 ms after the onset of handgrip cue sounds. Statistical parametric maps are superimposed on surface-rendered high-resolution MRIs. The color bar indicates T-values.

Although the ERD analyses for alpha frequency band in the motivation session relative to the control session at the voxel level did not show that the dorsolateral prefrontal cortex reached a statistically significant level, the ERD was statistically significant at the cluster level in the right dorsolateral prefrontal cortex (x = 47, y = 43, z = 10; inferior frontal gyrus; Brodmann's area 46; T-value = 4.99; Figure 5) within the time window of 400 to 500 ms after the onset of handgrip cue sounds.

**Figure 6:**
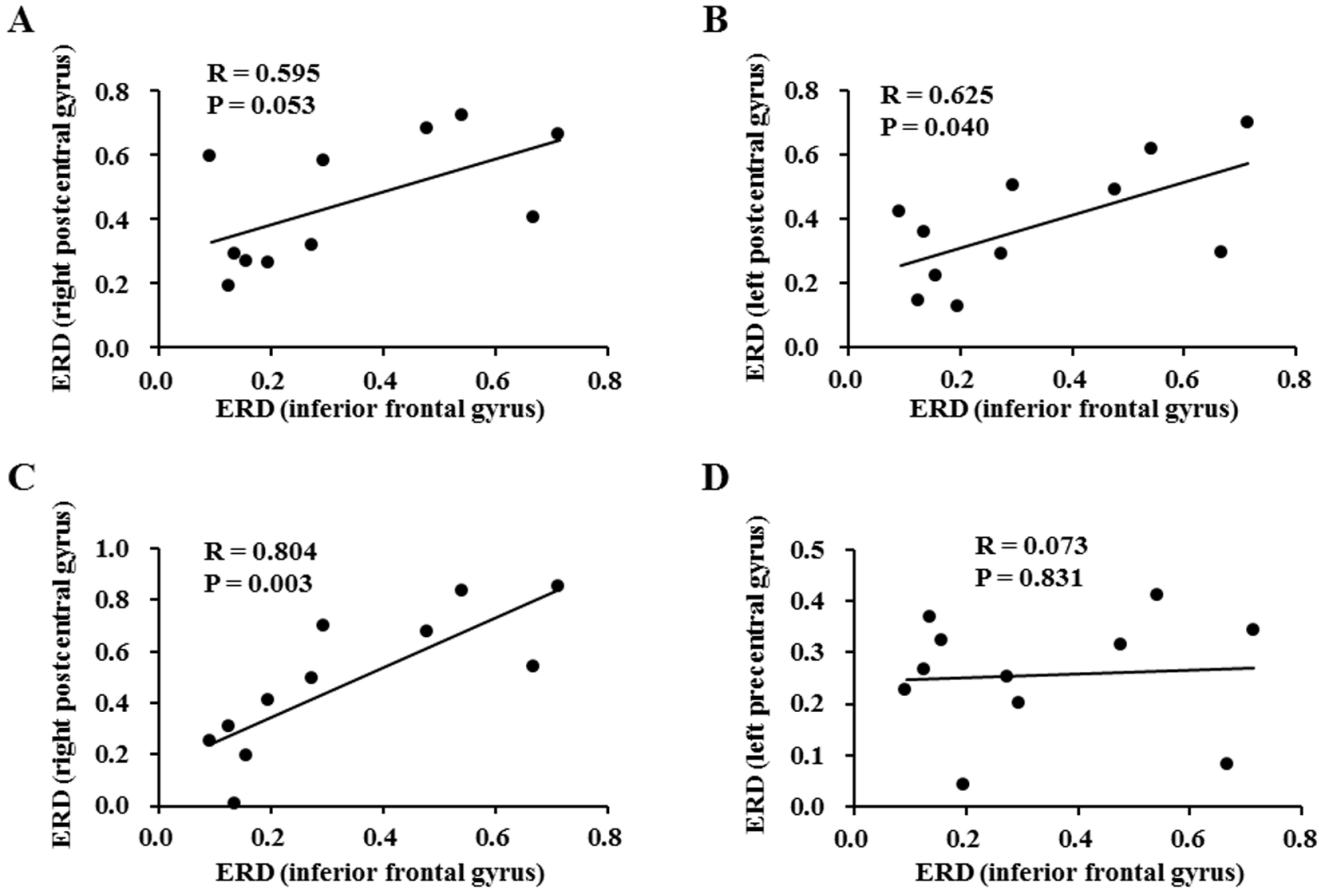
Relationships between the event-related desynchronization (ERD) level of alpha frequency band in the inferior frontal gyrus (Brodmann's area 46) and those in the right postcentral gyrus (time window of 500–600 ms after the onset of handgrip cue sounds) (A), left postcentral gyrus (500–600 ms) (B), right postcentral gyrus (600–700 ms) (C), and left precentral gyrus (800–900 ms) (D) (n = 11). Linear regression lines, Pearson's correlation coefficients, and P values are shown.

To evaluate the relationships between the ERD level of alpha frequency band in the right dorsolateral prefrontal cortex and those in the sensorimotor areas, correlation analyses were performed. The ERD level in the prefrontal cortex had a trend toward a positive correlation with that in the right sensorimotor area (R = 0.595, P = 0.053; time window of 500 to 600 ms [Figure 6A]) and that level was positively associated with those in the left (R = 0.625, P = 0.040; time window of 500 to 600 ms [Figure 6B]) and in the right (R = 0.804, P = 0.003; time window of 600 to 700 ms [Figure 6C]) sensorimotor areas. The ERD level in the prefrontal cortex was not associated with that in the contralateral M1 [R = 0.073, P = 0.831; time window of 800 to 900 ms (Figure 6D)]. However, the ERD level in the left M1 had a trend toward positive a association with that in the left sensorimotor area (R = 0.534, P = 0.074; time window of 500 to 600 ms).

## Discussion

In this MEG study, alpha-band ERDs of the motivation session relative to the control session within the time windows of 500 to 700 and 800 to 900 ms after the onset of handgrip cue sounds were identified in the sensorimotor areas (Fig. 4 and Table 1). In addition, the alpha-band ERD within the time window of 400 to 500 ms was identified in the right dorsolateral prefrontal cortex (Brodmann's area 46) (Fig. 5). The ERD level in the right dorsolateral prefrontal cortex was positively associated with that in the sensorimotor area within the time window of 500 to 700 ms (Fig. 6). These results indicate that the right dorsolateral prefrontal cortex is involved in the neural substrates of the facilitation system and activates the sensorimotor areas to overcome central fatigue.

We tried to identify the neural substrates of the facilitation system during physical fatigue by comparing neural activities during imagery of maximum handgrips between motivation and control sessions, as the difference between these conditions is limited to the presence or absence of the enhanced facilitation system (Fig. 3). Our MEG study showed that the sensorimotor areas and the right dorsolateral prefrontal cortex (Brodmann's area 46) were specifically activated during the motivation session (Figs. 4 and 5 and Table 1), suggesting that these brain regions are involved in the neural substrates of the facilitation system during physical fatigue. Because the neural activity in the right dorsolateral prefrontal cortex during imagery of maximum handgrips showed positive associations with that in the sensorimotor areas (Fig. 6) and activation in the dorsolateral prefrontal cortex proceeded that in the sensorimotor areas (400 to 500 ms vs. 500 to 900 ms; Figs. 4 and 5), the dorsolateral prefrontal cortex seems to play a primary role in increasing motor output from the contralateral M1 (Fig. 4C). The role may be through activation of the sensorimotor areas (Fig. 4A and B) in order to compensate for central fatigue.

Recently, we reported that after a fatigue-inducing maximum handgrip session, while the level of the motor movement-evoked MEG response in M1 was decreased, that in the ipsilateral area was increased, and activation levels in the contralateral and ipsilateral M1 were both positively associated with those in the dorsolateral prefrontal area [6]. Because the dorsolateral prefrontal cortex alters motor output from M1 [22]–[24] and the endurance to carry out a physical task (duration) was related to activation in the dorsolateral prefrontal cortex [7], this brain area may attempt to compensate for the loss of the force-generating ability of fatiguing muscles and even compete with the inhibitory input to the sensorimotor area by recruiting the descending motor output [6]. Although ventrolateral prefrontal cortex, by driving the motor cortex, was reported to constitute a brain pathway that allows emotional arousal to facilitate physical effort [25], we did not show that this brain is involved in the neural substrates of the facilitation system, maybe because of methodological differences.

There are four limitations to our study. First, we performed our study with a limited number of participants. To generalize our results, studies involving a large number of participants are essential. Second, it was difficult to assess the neural activities of the brain regions located deeply by using MEG. Therefore, some brain regions involved in the neural substrates of facilitation system might be missed because of the limitations of MEG. For example, the basal ganglia and orbitofrontal cortex are considered to be associated with the facilitation system under the condition of physical fatigue [6], [7], [26]–[28]. Future studies using other neuroimaging techniques, such as functional magnetic resonance imaging and positron emission tomography, would address this limitation. Third, the possibility that the difference in imagery of maximum handgrips between the motivation and control sessions represents imagery of strong vs. weak force production rather than the additional recruitment of the facilitation system. Forth, it is impossible to conclude with certainty from our results that the right dorsolateral prefrontal cortex is involved in the neural substrates of the facilitation system and activates the sensorimotor areas to overcome central fatigue. Causal data analyses linking brain activity in the right dorsolateral prefrontal cortex with activity in the sensorimotor areas would be necessary to draw this conclusion.

In conclusion, our results suggest that the right dorsolateral prefrontal cortex is involved in the neural substrates of the facilitation system and increases motor output of the contralateral M1 through activation of the sensorimotor areas during physical fatigue. Dysfunction of the facilitation system has been considered to play a pivotal role in the pathophysiology of motor-related chronic fatigue in multiple sclerosis and chronic fatigue syndrome, and this dysfunction may be a common feature of motor-related fatigue in human diseases or syndromes [28]–[30]. Our findings provide new perspectives on the neural mechanisms underlying physical fatigue as well as on the pathophysiology of chronic fatigue in human diseases or syndromes.
